# Hydrogel Containing Solid Lipid Nanoparticles Loaded with Argan Oil and Simvastatin: Preparation, In Vitro and Ex Vivo Assessment

**DOI:** 10.3390/gels8050277

**Published:** 2022-04-29

**Authors:** Muhammad Farhan Ali Khan, Asim Ur.Rehman, Haidar Howari, Aiyeshah Alhodaib, Faiz Ullah, Zia ul Mustafa, Abdelhamid Elaissari, Naveed Ahmed

**Affiliations:** 1 Department of Pharmacy, Faculty of Biological Sciences, Quaid-i-Azam University, Islamabad 45320, Pakistan; farhanali@bs.qau.edu.pk (M.F.A.K.); arehman@qau.edu.pk (A.U.); 2 Department of Physics, Deanship of Educational Services, Qassim University, Buraydah 51452, Saudi Arabia; haidarh1@yahoo.com; 3 Department of Physics, College of Science, Qassim University, Buraydah 51452, Saudi Arabia; 4 Department of Chemistry, Quaid-i-Azam University, Islamabad 45320, Pakistan; faizullah@chem.qau.edu.pk; 5 Punjab University College of Pharmacy, University of the Punjab, Lahore 54000, Pakistan; zia.ucp@gmail.com; 6 Univ Lyon, University Claude Bernard Lyon-1, CNRS, ISA-UMR 5280, 69622 Villeurbanne, France; abdelhamid.elaissari@univ-lyon1.fr

**Keywords:** hyperlipidaemia, simvastatin, improved therapeutic efficiency, transdermal hydrogel

## Abstract

Transdermal hydrogels have the potential to improve therapeutic outcomes via enhancing bioavailability and reducing toxicity associated with oral delivery. The goal of the present study was to formulate and optimise argan oil loaded transdermal hydrogel containing lipid nanoparticles. The high pressure homogenization (HPH) method was utilised to fabricate Simvastatin loaded solid lipid nanoparticles (SIM-SLNs) with precirol ATO 5 as a lipid core and Poloxamer 407 (P407) to stabilise the core. The optimised nanoformulation was characterised for its particle diameter, zeta potential, surface morphology, entrapment efficiency, crystallinity and molecular interaction. Furthermore, transdermal hydrogel was characterised for physical appearance, rheology, pH, bio adhesion, extrudability, spreadability and safety profile. In vitro and ex vivo assays were executed to gauge the potential of SLNs and argan oil for transdermal delivery. The mean particle size, zeta potential and polydispersity index (PDI) of the optimised nanoparticles were 205 nm, −16.6 mV and 0.127, respectively. Crystallinity studies and Fourier transform infrared (FTIR) analysis revealed no molecular interaction. The in vitro release model explains anomalous non-Fickian release of drug from matrix system. Ex vivo skin penetration studies conducted through a fluorescence microscope confirmed penetration of the formulation across the stratum corneum. Hydrogel plays a crucial role in controlling the burst release and imparting the effect of argan oil as hypolipidemic agent and permeation enhancer.

## 1. Introduction

Hyperlipidaemia is a well-known risk factor for cardiovascular mortality and morbidity. It mainly causes atherosclerosis, which in turn leads to coronary heart disease [1]. It is usually characterised by elevated levels of low-density lipoproteins and triglycerides. Escalated interest in argan oil, obtained from *Argania spinose*, is due to its antioxidant properties. Individuals consuming argan oil are less prone to heart attacks as compared to non-users [2]. It is also a hepatoprotective and choleretic agent that prevents atherosclerosis and hypercholesterolemia [3]. Simvastatin (SIM) is an anti-hyperlipidaemic drug having low bioavailability (<5%), poor aqueous solubility (30 µg/mL) and a high partition co-efficient (log P~4.5) [4]. It is a competitive inhibitor of 3-hydroxy-3-methylglutaryl coenzyme A (HMG-CoA) reductase and is mainly metabolised in the gut and liver by the cytochrome 3A system, which presents a major obstacle in the development of oral dosage forms. This route of administration also causes gastric lesions such as ulcers and erosions. Intramuscular, subcutaneous and intravenous routes of administration can cause discomfort to the patient as well as irritation at the site of injection and local soft tissue damage [5].

To circumvent issues of poor bioavailability and Gastrointestinal tract (GIT) irritation, conventional approaches are not enough [6]. Hence, there is a dire need to develop a novel dosage form that can combat the problems of poor water solubility and excessive metabolism [7]. A transdermal delivery system can reduce various side effects associated with oral dosage forms [8]. Incorporating SLNs into the transdermal hydrogel offers several benefits: lack of bio-toxicity, sustained drug release, better physical stability, high drug incorporation, improved bioavailability and reduced side effects [9,10]. Based on the lipophilic nature of SIM, lipid nanoparticles loaded into transdermal hydrogel containing argan oil might be a promising strategy to overcome the problem of low oral bioavailability and to provide synergistic hypolipidemic activity.

In recent decades, nanoparticulate carrier systems have been investigated to modulate pharmacokinetics, physicochemical properties, biocompatibility, therapeutic efficiency and bioavailability of poorly aqueous soluble drugs [11]. SLNs have gained augmented interest due to their superior characteristics compared to their colloidal counterparts [12,13]. Lipophilic drugs can be incorporated more effectively with higher encapsulation efficiency, providing a wide range of applications [14]. Encapsulation of drug molecules within this lipid matrix can prevent degradation and also impart prolonged release properties [15]. Moreover, macro molecules such as oligonucleotides, proteins, DNA and RNA can also be delivered through this carrier system [16]. Drugs incorporated into lipid nanoparticles exhibit better permeation as compared to conventional dosage forms [17]. Glycerides have created considerable interest in the development of controlled release dosage forms. Precirol ATO 5, also known as glyceryl palmitostearate, imparts prolonged release properties [18]. P-407 is a non-ionic surfactant which is used for sustained drug delivery. According to the FDA, it enhances stabilization; thus, nanoparticles can be optimised using this surfactant [19].

The objective of current study is to evaluate characteristics of SIM-SLNs based transdermal hydrogel. To fabricate SIM-SLNs, a HPH technique was used, with a lipid core stabilised by a surfactant mixture, and they were characterised for in vitro release behaviour and physicochemical properties after loading into hydrogel. Finally, a transdermal drug delivery system was developed to achieve sustained release and enhanced permeation using argan oil as a permeation enhancer. Following transdermal administration of SIM-SLNs to rat models, various pharmacokinetic parameters were also calculated.

## 2. Materials and Methods

### 2.1. Materials

Precirol ATO 5 was a generous gift from Gattefosse France. SIM and P407 (Sigma-Aldrich, Steinheim, Germany), Ethanol and Carbopol 934 were acquired from BDH Laboratory Supplies. Argan oil was purchased from Laboratories Omega Pharma, Chatillon, France. All other chemicals used in the current study were of analytical grade and were used without further processing.

### 2.2. Methods

#### Preparation of SIM-SLNs

The SLNs were prepared by the HPH technique with slight modifications [20]. Briefly, 50 mg of precirol ATO 5 was completely dissolved in 2 mL of ethanol by heating at 60 °C in a water bath. The resultant organic phase (clear appearance) was quickly mixed with an aqueous phase (20 mL) containing surfactant (0.5%) preheated to the same temperature. This mixture was homogenised through a homogenizer (D-91126 Heidolph instruments, Schwabach, Germany) at 24,500 rpm for 5 min at 60 °C. Then, the obtained SLNs were cooled in an ice water bath to quickly crystallise the lipid. Drug-loaded SLNs were fabricated through the same method by adding SIM to the organic phase. For optimization of formulation (SIM-SLNs), a trial-and-error method was used, varying the concentration of different components such as Precirol and P-407. An optimised formulation (H) was selected on the basis of smaller particle size and higher encapsulation efficiency. The resultant trials for optimization are tabulated in Table 1.

### 2.3. Physicochemical Characterization of Nanoparticles

#### 2.3.1. Particle Size, Polydispersity Index, Zeta Potential and Entrapment Efficiency

Photon correlation spectroscopy and electrophoretic light scattering techniques were used to determine particle size, polydispersity index (PDI) and zeta potential of the samples through zetasizer Nano ZS 90 (Malvern Instruments; Worcestershire, UK), equipped with software (version 6.34) and a He-Ne laser at a wavelength of 635 nm and static scattering angle of 90 degrees. Briefly, 10 µL of the sample was mixed with 1 mL of deionised water and vortexed for 2 min, followed by analysis with a zetasizer. Each result displayed was measured in triplicate.

Spectrophotometric analysis was used to determine the amount of SIM incorporated in SIM-SLNs. For determination of entrapment efficiency, formulation (1 mL) was taken in a falcon tube and volume was made up to 10 mL with ethanol. After that, the mixture was centrifuged for 1 h at 13,500 rpm in a centrifuge (Hermle Labortechnik, Wehingen, Germany). Supernatant (100 µL) was taken for further processing and diluted with 10 mL of ethanol. For determination of drug content, a UV-visible spectrophotometer (Dynamica, Halo DB-20, Livingston, UK) was utilised at λ_max_ of 239 nm.

#### 2.3.2. Scanning Electron Microscopy (SEM)

SIM-SLNs were evaluated for surface morphology using a Scanning electron microscopy (Tescan vega-3, model imu vp-sem, New York, NY, USA). For this purpose, nanoparticles were lyophilised with the help of a lyophiliser (Alpha 1-2 LD plus, Christ, Osterloh, Germany) to obtain dried particles. High resolution SEM was used to analyse the surface properties, structure and size of dried particles [21].

#### 2.3.3. Fourier Transform Infrared Spectroscopy (FTIR)

FTIR analysis was carried out to investigate any potential interaction among the individual components of the formulation. The FTIR spectra of SIM, Precirol, SIM-SLNs, and P407 were acquitted between 4000 cm^−1^ and 450 cm^−1^ using an FTIR spectrophotometer (L16000A, Perkin-Emler USA). Each 10 mg sample was triturated with 100 mg of KBr (potassium bromide), and all measurements were executed in triplicate at room temperature [22].

#### 2.3.4. Powder X-ray Diffractometry (PXRD)

The X-ray diffraction patterns of lyophilised SIM-SLNs and their individual solid components (SIM, Precirol, and Poloxamer) were studied by a powder X-ray diffractometer (Rigaku Inc.,Tokyo, Japan) using a copper anode as a source of radiation. Scanning of samples was performed at room temperature (20 kV, 5 mA). After that, absolute intensity was recorded in the range of 5–80 against theta 2 and a scanning rate of 1.2°/min [23].

### 2.4. Formulation of SIM-SLNs Loaded Transdermal Hydrogel

**Scheme 1:**
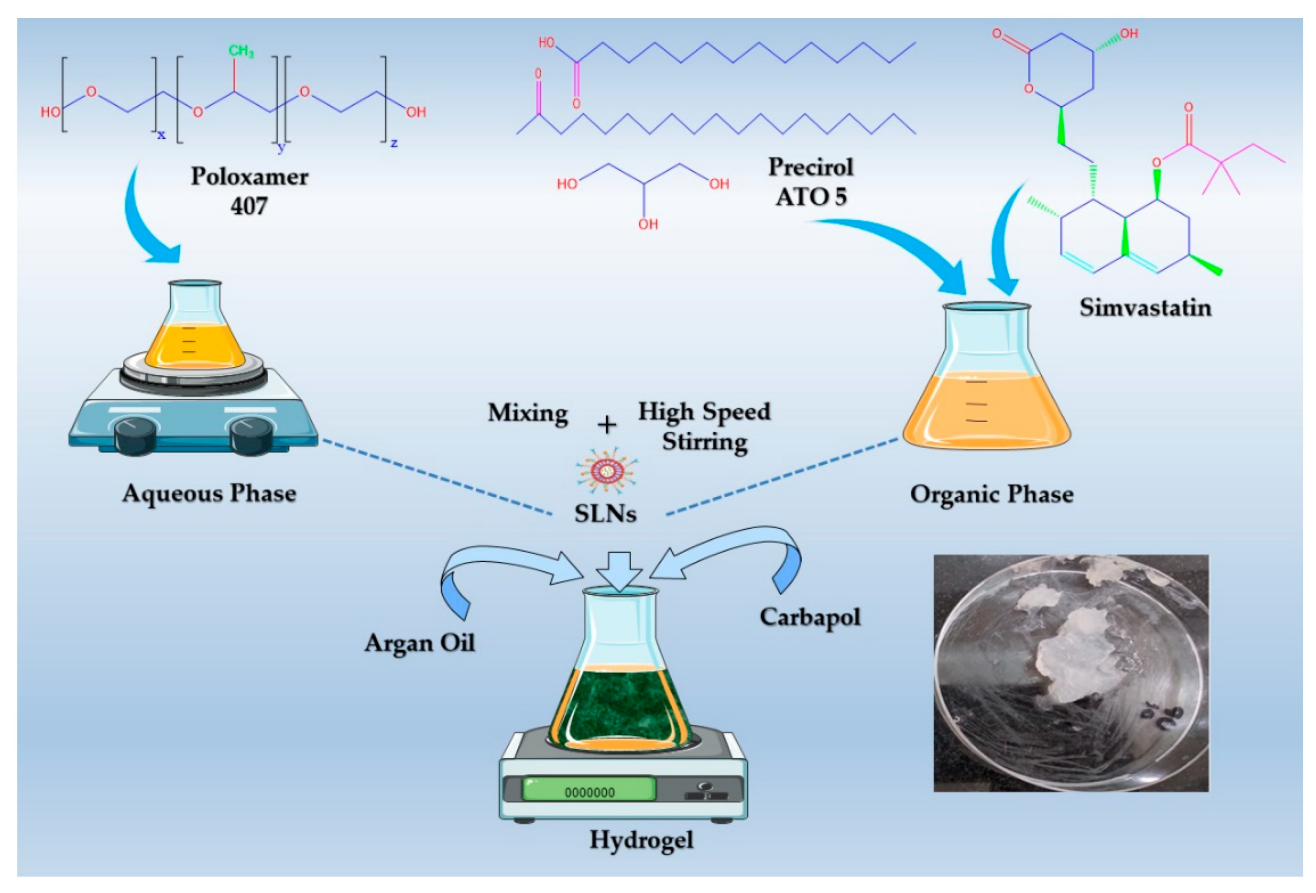
Illustration of hydrogel formation.

Carbopol 934 was utilised for the preparation of the transdermal hydrogel (Scheme 1). Briefly, a 1% carbopol solution was prepared by dissolving it in distilled water with stirring at 300 rpm, followed by SIM-SLN addition to the solution. Glutaraldehyde (0.5%) was added as a cross linker during stirring. At the end, triethanolamine (0.5%) as a gelling agent was added to neutralise the pH of the medium. The hydrogel having a penetration enhancer was prepared in the same manner by adding argan oil (1 mL) before incorporation of triethanolamine [24].

### 2.5. Characterization of Transdermal Hydrogel

#### 2.5.1. Physical Appearance and pH Measurement

The pH of the transdermal hydrogel was measured by utilising a pH metre (PHS25CW Bante instrument, Chicago, IL, USA). Physical appearance includes smoothness, transparency, homogeneity and colour. The SIM-SLNs hydrogel was also inspected for these characteristics [25].

#### 2.5.2. Rheological Study and Spreadability of Transdermal Hydrogel

Brookfield viscometer (DV-I Prime Brookfield engineering laboratories, Inc., 11 Commerce Boulevard, Middleboro, MA, USA) with S63 spindle was used to measure the viscosity of a hydrogel at various shear rates. For the purpose, 2 g of hydrogel was taken in a cone and viscosity was measured at 0, 0.5, 1, 2.5, 4, 5, 10, 20, 50 and 100 rpm [26].

The glass slide method was utilised for the measurement of hydrogel spread ability. A glass slide was marked with a circle of 1 cm having 0.5 g of gel placed in the centre [27]. Another glass slide was placed on it in a manner that hydrogel was sandwiched between two glass slides. After that, a 500 g weight was placed on the glass slides, and the increment in diameter was measured. Using Equation (1), results were obtained with respect to applied mass and spreading area [28].

(1) $$Spreadability\;index\;(Si)=d^{2}\;\times \;\frac{\pi }{4}$$

#### 2.5.3. Swelling index, Bio-adhesion and Extrudability

In order to measure the swelling index of the hydrogel, the gravimetric method was used. For this purpose, transdermal hydrogel was lyophilised and then placed in a phosphate buffer at pH 5.5. Swollen hydrogel was taken out of the medium and weighed at different time intervals (0.5, 1, 1.5, 2, 2.5, 3, 3.5 and 4 h) until weight became constant. Percentage swelling was measured using Equation (2).

(2) $$Swelling\;ratio=\frac{w2-w1}{w1}\;\times \;100$$

A disintegration apparatus was used to determine the bio adhesion time. An apparatus beaker (500 mL) was filled with phosphate buffer of pH 5.5 at 32 °C. Rat skin was attached with a glass slide and then was placed on the disintegration apparatus with the help of adhesive tape. In the next step, a transdermal hydrogel was applied to the rat’s skin. After immersion into a phosphate buffer solution, the whole assembly was allowed to move up and down. The time it took to remove the applied hydrogel was recorded accordingly [29].

For determination of extrudability, an observation test was performed to assess expulsion of hydrogel from a tube. A closed aluminium collapsible tube was filled with 10 g of hydrogel, and a clamp was applied for prevention of any roll back. The cap was removed after placing a 500 g weight on the tube. The amount of hydrogel extruded was collected, weighed and the percentage was calculated [30].

### 2.6. In Vitro Drug Release Studies

A dialysis membrane was used to perform in vitro release studies in phosphate buffer at pH 7.4 and 5.5. The formulation was placed in a dialysis sac to assess drug release at various time intervals. After dipping a dialysis sac in a beaker, this medium was placed on a shaking water bath at 37 °C. All studies were performed in triplicate [31]. Sampling time was 0.25, 0.5, 1, 3, 6, 12, 24 and 48 h. At each time interval, 1 mL of sample was withdrawn. Phosphate buffer of the respective pH was used to replace the amount of sample withdrawn to maintain the sink conditions. A UV-visible spectrophotometer was utilised to quantify these samples at a wavelength of 239 nm. To calculate drug release, a graph was plotted between percent drug release versus time using MS Excel. First order, zero order, Higuchi, Hixon–Crowel, and Korsmeyer–Peppas kinetic models were applied for evaluation of the release profile of SIM-SLNs and SIM-SLNs hydrogel.

### 2.7. Permeation Studies

Permeation studies were performed using rat skin. A healthy animal was sacrificed, and the skin from the abdominal portion was separated. After that, a surgical blade was used to remove all the hairs from the rat’s skin. Afterwards, the skin was placed in warm water to remove adipose tissue and stored in a freezer at −30 °C. To check permeation, it was placed in a buffer solution for half an hour before the experiment. Rat skin was utilised to check the permeability of SIM-SLNs through skin. The first one was a hydrogel having argan oil as a permeation enhancer, and the second was SIM-SLNs. A skin layer was placed on the donor compartment, and the formulation was applied to the skin. The receiver compartment was filled with phosphate buffer solution (pH 7.4), and samples were withdrawn with long needle syringes. The sampling interval was 0.25, 0.5, 1, 3, 6, 12 and 24 h. A sample (1 mL) was withdrawn from the receiver compartment after each interval, and sink conditions were maintained. The absorbance values of all samples were recorded using a UV-visible spectrophotometer at 239 nm. The study was performed in triplicate, and Microsoft Excel was used to interpret the results.

### 2.8. Ex Vivo Penetration Analysis through Fluorescent Microscope

A fluorescent microscope was utilised to study the distribution of nanoparticles in rat skin. Rhodamine-loaded nanoparticles were formulated by injecting the dye (2 mg) into the organic phase. Nanoparticles were centrifuged at 13,500 rpm for 1 h to separate un-entrapped dye. Entrapped Rhodamine-loaded nanoparticles were collected from the pellet and incorporated into the hydrogel. Rat skin was placed on the Franz diffusion cell for 5 h with the application of a hydrogel formulation. After that, the transactional part of rat skin was obtained with the help of a cryostat microtome. Images were visualised with the help of a fluorescent microscope by keeping a blank skin as a control [32].

### 2.9. Evaluation of Skin Structure after Treatment with Hydrogel

In order to evaluate the effect of the permeation enhancer, skin structure after application of the hydrogel was investigated with Fourier transform infrared spectroscopy (FTIR) because it is supposed that permeation enhancers may induce structural changes in the stratum corneum. Rat skin was placed on the Franz diffusion cell and treated with hydrogel for 5 h. After that, the skin was washed and subjected to FTIR analysis, while untreated rat skin was used as a control [33].

### 2.10. Stability Studies

According to the ICH guidelines, stability studies were carried out for transdermal hydrogel. Accelerated studies were performed for a period of six months (1, 3 and 6 months) at a temperature of 25 °C and a relative humidity of 60% [34]. Hydrogel was evaluated on the basis of changes in pH and physical appearance.

## 3. Results and Discussion

### 3.1. Preparation and Physicochemical Properties of SIM-SLNs

SIM-SLNs were successfully prepared by HPH technique with good reproducibly. Precirol ATO 5 was used as a solid lipid core of SIM-SLNs, stabilised by surfactant. Precirol is a pharmaceutically acceptable, biocompatible and biodegradable lipid having high incorporation efficiency and stable lipid core with sustained drug release. P-407, a non-ionic surfactant, was used to interact and form a miscible surfactant shell with Precirol core. The surfactant provides steric stabilization to improve the stability of SIM-SLNs by reducing surface interactions between the particles [35,36]. After analysing all stable formulations, optimised formulation (H) was selected on the basis of smaller particle size and maximum entrapment efficiency (Table 2). An increase in particle size was observed when a large amount of Precirol ATO 5 was used. In one-way ANOVA analysis, it is evident that particle size (F = 13.204, *p* = 0.001) and PDI (F = 8.608, *p* = 0.004) were significantly associated with lipid concentration as particle size and PDI increase by increasing lipid concentration (Table 2). This illustrates the fact that the size of SLNs depends upon concentration of lipids because lipids have the tendency to coalesce at high concentrations [37]. Increase in particle size can be due to density difference between external and internal phases, or it may occur due to a reduced diffusion rate of solute molecules in the outer phase. It was observed that increasing concentration of surfactant causes decrease in particle size. It might be due to reduction in surface tension between the organic and aqueous phases [38].

**Figure 1:**
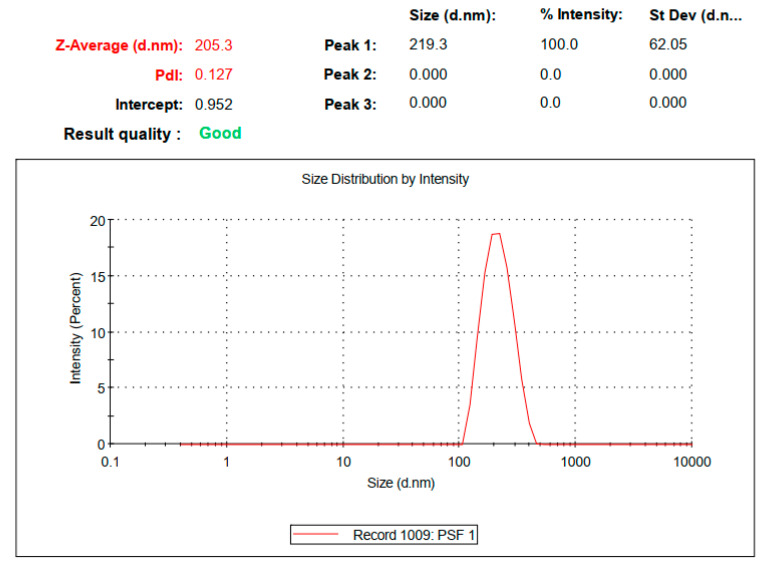
Particle size distribution of optimised drug loaded formulation of SLNs.

Blank and drug loaded nanoparticles exhibited particle sizes of 173 and 205 nm with PDI values of 0.177 and 0.127 expressing narrow size distribution (Figure 1). The phenomenon of size increase is commonly observed after drug incorporation. The particle size plays a crucial role in the uptake of nanoparticles after transdermal administration. Nanoparticles smaller than 300 nm are desirable for effective transdermal transport into the systemic circulation [39].

PDI value below 0.3 indicates homogenous distribution; hence, it is evident that SIM-SLNs were uniform in size [40]. The zeta potential of the nanoparticulate formulation is a key indicator for stability influencing physical stability of colloidal dispersions. Due to electrostatic repulsion between similarly charged particles, higher zeta potential prevents aggregation thus conferring stability to colloidal dispersion. SIM-SLNs exhibited a zeta potential of about −16.6 mV which ensures electrostatic stabilization [41]. SLNs prepared by the HPH method displayed a smaller particle size (205 nm), low Polydispersity index (0.127) and zeta potential (−16.6 mV), which warrants physical stability of formulation [42]. These results also augmented in linear regression models that indicates lipid concentration is a significant determinant of particle size and PDI, and any increase in lipid concentration results into increase in particle size (β = 7.953, *p* < 0.05) and PDI (β = 0.011, *p* < 0.05). While increase in Poloxamer concentration is negatively related to particle size and PDI, as increase in surfactant concentration leads to decrease in particle size (β = −57.675, *p* > 0.05) and PDI (β = −0.127, *p* > 0.05).

Encapsulation efficiency is a parameter of interest to deliver drug in higher dose more precisely at site of action. Entrapment efficiency is bound to increase with increasing concentration of lipid from 74% to 82% as shown in Table 2. The lipid acts a solubilizing agent for highly lipophilic drugs. Use of solid lipids cause enormous defects in crystal lattice resulting higher imperfections, thus, enhancing the space to entrap drug molecules [43]. As compared to solvent emulsification method, use of the HPH technique leads to higher entrapment efficiency and enhanced percentage yield [44]. One-way ANOVA analysis revealed that entrapment efficiency was increased by increase in lipid concentration (Table 2). Entrapment efficiency is bound to increase with the increasing concentration of lipid because it acts as a solubilizing agent for highly lipophilic drugs. Linear regression analysis also indicates any increase in lipid concentration results into increase in EE (β = 0.437, *p* < 0.05). Results also indicate that poloxamer concentration had a significant association with entrapment efficiency; by increasing poloxamer concentration, there is significant decrease in entrapment efficiency (F = 3.848, *p* = 0.043). This is also evident in linear regression as increase in surfactant concentration is negatively related to entrapment efficiency (β = −6.727, *p* > 0.05).

### 3.2. Particle Morphology

**Figure 2:**
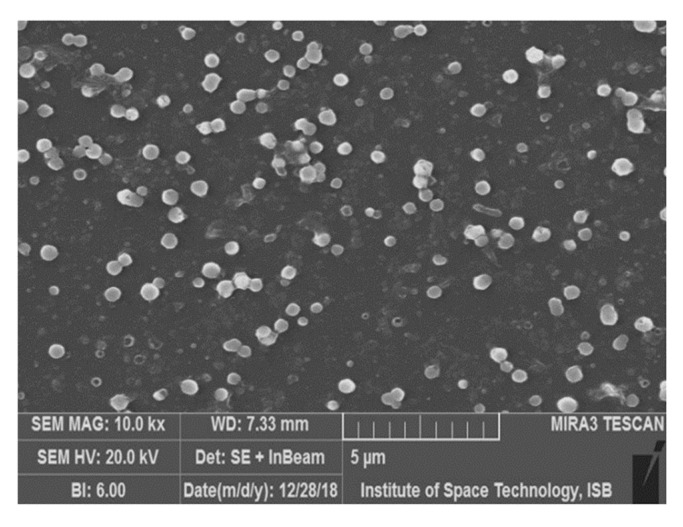
Scanning electron microscope (SEM) image of Precirol lipid nanoparticles with spherical morphology loaded with SIM.

Scanning electron microscopy (SEM) was utilised to determine the morphology of blank and drug loaded SLNs. Figure 2 exhibits SEM images at different intensifications. Results confirm the sphericity of nanoparticles. SEM images of SIM-SLNs exhibited the uniform and spherical nature of the nanoparticles. It was noted that results are in agreement with earlier studies [45].

### 3.3. FTIR Analysis

**Figure 3:**
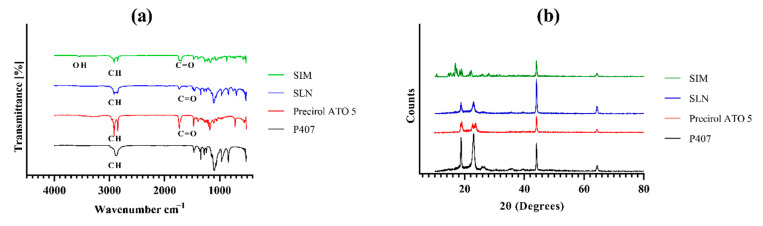
(**a**) FTIR and (**b**) PXRD spectrum of SIM, SLN formulation, Precirol ATO 5 and P407.

FTIR analysis was performed to investigate molecular interaction between drug and lipid matrix. The spectra of SIM exhibited characteristic peaks at 3545 cm^−1^ due to stretching vibration of the OH groups, and Peaks at 2914 cm^−1^ and 2849 cm^−1^ were due to stretching vibration of the CH group (Figure 3). Stretching vibrations of the ester group showed peaks at 1728 cm^−1^. The same characteristic peaks were observed in the spectra of drug loaded SLNs which confirms chemical stability of SIM in SLNs formulation. FTIR analysis confirmed that there was no chemical or electrostatic interaction between ingredients of the nanoparticulate formulation. Absence of any new peak or functional group also exhibits absence of any interaction among constituents. SLNs prepared through this technique exhibit regular bands in FTIR spectra. While SLNs prepared through other techniques such as solvent evaporation show significant convolution in FTIR spectra. Other studies also reported similar conclusion from FTIR analysis [46]. SIM characteristic peaks of FTIR can be seen in the formulation indicating no change in chemical structure of drug. Absence of OH stretch can be explained by intermolecular hydrogen bonding [47].

### 3.4. Degree of Crystallinity of SIM-SLNs

The crystalline behaviour of lyophilised SIM-SLNs was evaluated by using PXRD. X-ray diffraction analysis showed a number of sharp peaks demonstrating the crystalline nature of the drug (Figure 3). SIM exhibits sharp peaks in range of 10–30 which confirms the crystalline nature of drug, but after loading into lipid (Precirol), there were no characteristic peaks observed within this range. However, Precirol retained its crystalline form before and after drug loading. These results are in agreement with earlier study because the nature of drug changed from crystalline to amorphous while no change was observed in its lipid nature. The decrease in crystallinity might be due to absorption of the surfactant on SLNs surface. Results are in harmony with previous studies suggesting an amorphous state of SIM in the solid lipid core [48].

### 3.5. Physicochemical Evaluation of Transdermal Hydrogel

For the development of the transdermal hydrogel, the barrier function of the stratum corneum is of prime importance. To transport SIM-SLNs across the stratum corneum, a Carbopol based hydrogel was formulated. Fabrication of a secondary carrier was essential to control the burst release and to impart the effect of argan oil as permeation enhancer and hypolipidemic ingredient. Furthermore, transdermal hydrogel also improves the patient compliance. To deliver the drug more efficiently through the transdermal route, permeation enhancers are used. Permeation enhancers should be non-reactive, non-toxic, biocompatible, etc. For this purpose, Argan oil was utilised as permeation enhancer to augment the permeation from the lipid system [49].

#### 3.5.1. Physical Appearance and pH of Formulation

The transdermal hydrogel was smooth in texture with no grittiness and was uniform and clear. Normal pH of skin ranges from 4–6 while pH of transdermal hydrogel was found to be 5.3 indicating slightly acidic nature. It is beneficial for transdermal formulation because pH at skin surface and within different layers remain acidic favouring intact delivery of nanoparticles across skin. pH is a significant parameter and should be within range (4–6); otherwise, it can cause skin irritation and incompatibility. Hence, formulations with pH falling in between this range are suitable for drug delivery through skin [50].

#### 3.5.2. Spreadability and Rheological Behaviour of Transdermal Hydrogel

In order to evaluate ease of application and consistency, spreadability measurement is an important parameter. Spreadability of drug loaded hydrogel was measured by using glass slide method. The value was found to be 273.2 ± 0.5 mm^2^. Ease of application and spreadability is an important property of formulation for uniform drug delivery. It also improves patient compliance. Spreadability studies confirmed ease of spreading on skin surface. These results are in accordance with previous studies as values are within an acceptable range [51].

**Figure 4:**
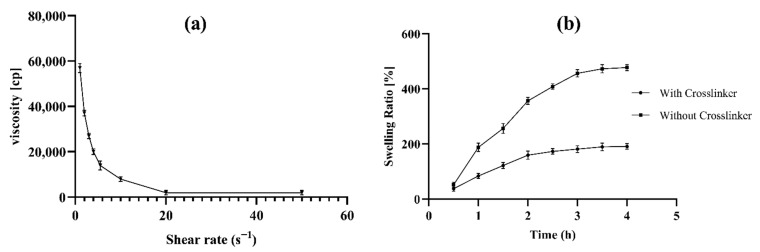
(**a**) Rheology of hydrogel; (**b**) swelling index of lyophilised hydrogel with and without cross linker.

Viscosity of transdermal hydrogel was determined at different shear rates as shown in Figure 4. It can be seen that there is an inverse relationship between viscosity of hydrogel and shear rate. Viscosity of transdermal hydrogel is going to be reduced by enhancing shear rate and vice versa. The behaviour of the hydrogel is similar to shear thinning gels, which behave like liquids under stress while converting to viscous form when the stress is removed. This kind of behaviour under stress conditions is known as shear thinning. Though the exact cause of shear thinning is not fully understood, it is widely regarded to be the effect of small structural changes within the fluid, such that microscale geometries within the fluid rearrange to facilitate shearing. For assessment of adhesive capability and retention time on skin, rheological properties are of prime importance. The thixotropic nature of the hydrogel has the benefit of easy application on skin requiring little force [52].

#### 3.5.3. Swelling index, Bioadhesion and Extrudability

Swelling studies of transdermal gel were performed with and without a cross linker. It was observed from swelling index results that a swelling ratio was higher in hydrogels having no cross linker. Significant reduction in swelling ratio was observed in hydrogels with cross linker (Figure 4). The reason might be that without cross linker, lipid chains create more space for diffusion of water in the gel matrix. In contrast, the cross-linked gel restricted the free movement of water, thus decreasing swelling ratio. It indicated that the cross linker imparts strength to the hydrogel and controls the burst release from hydrogel [53]. The retention time of the transdermal formulation was found to be 45 min. Slight decrease in retention time indicates presence of argan oil which reduces the viscosity. The reduction in bioadhesion might be due to the accelerated experimental conditions in the disintegration apparatus. The extrudability of Carbopol 934 based hydrogel was found to be 89% (8.9 g) which is a good property. The extrusion of hydrogel from the tube is an important parameter to enhance patient compliance and ease of application. In order to extrude gel from the tube, viscosity plays a significant role. Low viscous gels extrude more easily as compared to highly viscous gels. These results are in accordance with previous studies [30].

### 3.6. In-Vitro Release

**Figure 5:**
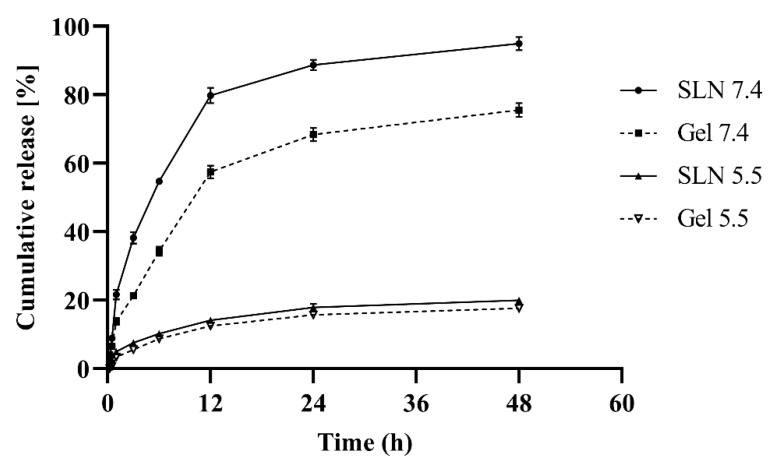
In vitro release profile of SLN formulation and Carbopol based hydrogel at pH 7.4 and pH 5.5.

A dialysis membrane method was adopted to evaluate drug release from SIM-SLNs at pH 7.4 and pH 5.5. Figure 5 illustrates that nearly 85% of all entrapped drug was released in 12 h from the SLNs formulation, while 65% of SIM was released from the transdermal hydrogel which indicates a more sustained release at pH 7.4. However, drug release was negligible at pH 5.5.

By applying various release models such as zero order, first order, Hixon–Crowel, and Korsmeyer–Peppas, the mechanism of drug release was determined. R^2^ values indicate that release from SIM-SLNs follows the Korsmeyer–Peppas model. The R^2^ value was 0.991. It also illustrates that release from transdermal hydrogel follows the Korsmeyer–Peppas model. The R^2^ value in this case is 0.989, and the n value was 0.651, indicating that drug release will follow anomalous non-Fickian release mechanism. Burst release from nanoparticles is a major concern for researchers all across the globe. This problem can be tackled by loading nanoparticles into secondary carriers: patches, films and gels. Keeping this in mind, Precirol based nanocarriers were successfully loaded into a Carbopol based hydrogel which is a secondary carrier. The burst release may be attributed to smaller particles with large surface area causing the fastest release at the initial stage. Moreover, burst release might be due to drug adsorbed onto or close to the SLNs surface [54]. To investigate the drug transport mechanism, the value of n in the Korsmeyer–Peppas model is important. Thus, n values between 0.5–1 correspond to anomalous non-Fickian diffusion and values below 0.5 show Fickian diffusion. Only the first 60% of the release is considered for estimation of this parameter [55]. An R^2^ value near to 1 explains that release of drug from nanoparticles follows a concentration dependent release (first order kinetics). On other hand, the hydrogel follows the Korsmeyer Peppas model R^2^ = (0.989). This model explains the anomalous non-Fickian release of drug from the matrix system [38].

### 3.7. Skin Permeability

**Figure 6:**
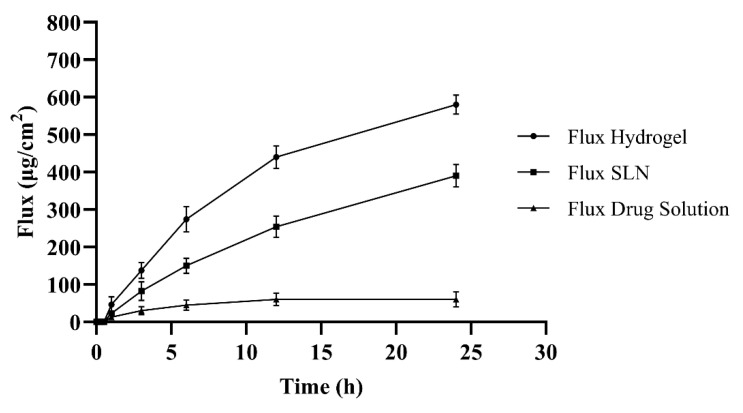
Ex vivo permeability studies of drug solution (control), SLNs and transdermal hydrogel at pH 7.4.

A Franz diffusion cell was utilised for determination of skin permeability. A comparison between permeability of transdermal hydrogel with and without permeation enhancer (argan oil) was also performed (Figure 6). A statistical test (ANOVA) was applied to determine permeability of these formulations. Enhanced skin permeability was observed in the transdermal hydrogel having permeation enhancer. The steady state flux (Jss) of the nanoparticles based transdermal hydrogel was measured. Jss of the hydrogel containing argan oil was higher (615 µg/cm^2^) as compared to the simple formulation (417 µg/cm^2^). It was concluded statistically (0.004) that hydrogel with permeation enhancer shows better permeability as compared to other formulations. It was concluded from permeation studies by applying statistical tool analysis of variance that a significant amount of drug permeated in the buffer medium of pH 7.4 through the skin. Increased permeation might occur due to the presence of argan oil which acts as a permeation enhancer. Most probably, the increase in permeation rate was attained due to the lipid coating and permeation enhancer. The skin permeation enhancement effect of argan oil is well known. It is composed of oleic and linoleic acid which enhances penetration through the intercellular pathway by interacting with the stratum corneum. Argan oil provides a dual benefit: it acts as a permeation enhancer by interaction with the skin lipid causing reversible fluidization, and also, it acts as an anti-hypercholesterolemic agent [55].

### 3.8. Ex Vivo Penetration Analysis through Fluorescent Microscope

**Figure 7:**
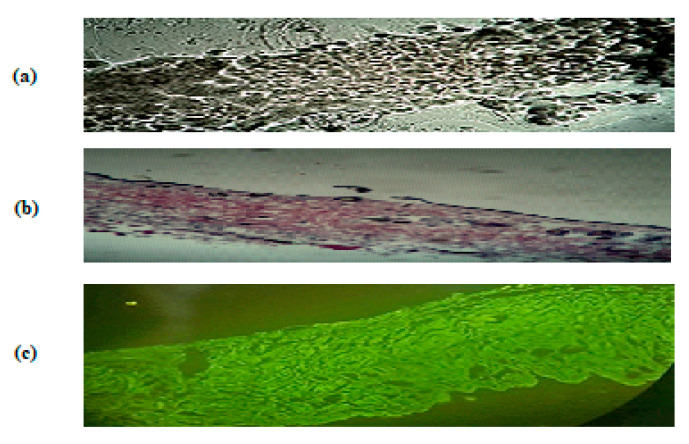
Fluorescence image of rat skin (transactional slice) after Rhodamine loaded nanoparticles permeation: (**a**) skin without treatment, (**b**) visualization under normal microscope and (**c**) green fluoresce exhibiting penetration across stratum corneum.

Fluorescent photomicrographs of rhodamine loaded nanoparticles illustrate the deeper penetration of nanoparticles as seen in the Figure 7. Incorporation of rhodamine resulted in strong fluorescent marking in the dermis layer. These results indicate nanoparticles deliver a substantial amount of drug across the skin. A fluorescence microscope (Leica mikroskopie & Systeme GmbH., Wetziar, Germany) was used to visualize the distribution of rhodamine loaded nanoparticles across the skin following the application of the hydrogel formulation with the permeation enhancer. It diffused into the deeper layers as can be seen from the green fluorescence. These results suggest that SLNs have the capability to enhance the penetration of SIM across the skin and hence are expected to enhance the bioavailability. These results are in accordance with previous results as they have also conducted permeation studies for SLNs [47].

### 3.9. Evaluation of Skin Structure after Treatment with Hydrogel

**Figure 8:**
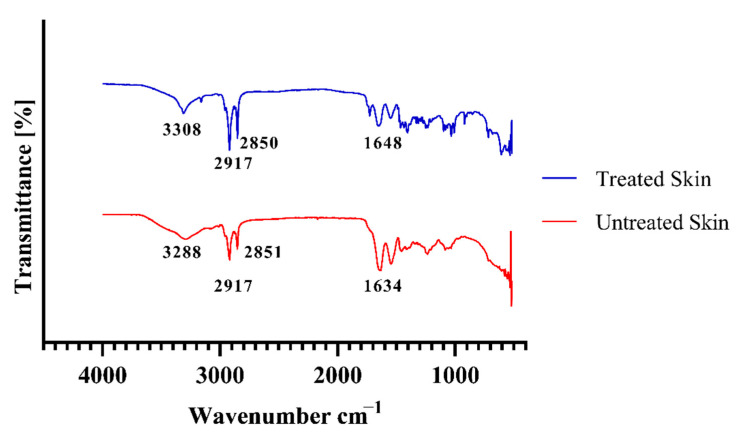
FTIR analysis of hydrogel treated skin and original skin without any specific treatment.

Permeation enhancers are known to reduce barrier function of stratum corneum by destabilizing and fluidizing skin lipids, hence enhancing percutaneous absorption. In this study, changes in epidermal structures were observed through FTIR. It was detected from the vibrations that there was a minute change in control and hydrogel NPs treated skin. The incorporation of permeation enhancer caused no damage to the skin as confirmed by the FTIR spectra (Figure 8). Small convulsions were observed in the spectra which may be due to argan oil induced intercellular penetration. However, these are minor changes, and skin has the ability to recover due to its regenerative and elastic properties. The present results suggest this formulation is a safe and promising transdermal delivery system.

### 3.10. Stability Studies of Hydrogel

Physical appearance of hydrogel exhibited no change in terms of phase separation, colour and grittiness at accelerated conditions as shown in Table 3. However, a slight increase in pH was observed from 5.3 to 5.6. Stability is an important parameter to ensure the effectiveness of formulation. The results of this study exhibited no gross change; thus, it can be considered that the hydrogel has a tendency to maintain its effectiveness.

## 4. Conclusions

Lipid nanoparticles have emerged as a versatile nano-platform for a number of applications in drug delivery. Hydrogels loaded with argan oil and NPs were successfully formulated with biodegradable and biocompatible ingredients for transdermal delivery. SIM-SLNs with uniform particle size distribution and spherical morphology demonstrated high encapsulation efficiency, better retention time, increased permeability and sustained in vitro drug release. Transdermal administration of nanoparticles via hydrogel might significantly enhance bioavailability. Consequently, argan oil based transdermal hydrogels containing SIM-SLNs could be promising nanocarriers to enhance therapeutic potential because argan oil acts as an antihyperlipidemic ingredient. The advantages of gels can be extended to those suffering from gastrointestinal problems or drugs having limited bioavailability. Further studies are required to confirm the synergistic effect of argan oil and SIM on the reduction of hyperlipidaemia.

## Figures and Tables

**Table 1:** Formulation of SLNs using HPH technique.

Formulation Code	Lipid Phase		Aqueous Phase (20 mL Water)
	Lipid (mg)	Solvent (mL)	P407 (%)
**A**	50	2	0.25
**B**	50	2	0.5
**C**	50	2	0.75
**D**	50	2	1
**E**	50	2	1.5
**F**	30	2	0.5
**G**	40	2	0.5
**H**	**50**	**2**	**0.5**
**I**	60	2	0.5
**J**	70	2	0.5

**Table 2:** Response parameters of formulation: entrapment efficiency, particle size and PDI.

Formulation Code	Entrapment Efficiency	Particle Size (nm)	PDI	Observation
**A**	65	230	0.400	Non uniform ^a^ and unstable ^b^
**B**	79	225	0.452	Uniform and stable ^c^
**C**	75	180	0.327	Uniform ^d^ and stable
**D**	68	175	0.252	Uniform and stable
**E**	63	166	0.174	Uniform and stable
**F**	74	177	0.268	Uniform and stable
**G**	77	182	0.372	Uniform and stable
**H**	**82**	**205**	**0.127**	**Uniform and stable**
**I**	79	241	0.407	Non uniform and stable
**J**	71	551	0.793	Non uniform and unstable

^a^ Globular appearance to naked eye. ^b^ Did not remain uniform for more than 90 days at 25 °C. ^c^ Remained uniform for more than 90 days. ^d^ Non globular appearance.

**Table 3:** Stability studies of hydrogel in terms of physical appearance and pH.

Time (Months)	Physical Appearance			pH
	Phase Separation	Color	Grittiness	
0	No	No change	None	5.3
3	No	No change	None	5.5
6	No	No change	None	5.6

## Data Availability

Not applicable.

## References

[1] Adi D., Abuzhalihan J., Wang Y.-H., Baituola G., Wu Y., Xie X., Fu Z.Y., Yang Y.N., Ma X., Li X.M. (2020). IDOL gene variant is associated with hyperlipidemia in Han population in Xinjiang, China. Sci. Rep..

[2] Asbahani A., El Miladi K., Badri W., Sala M., Addi E.H.A., Casabianca H., Mousadik A., El Hartmann D., Jilale A., Renaud F.N.R. (2015). Essential oils: From extraction to encapsulation. Int. J. Pharm..

[3] Badreddine A., Zarrouk A., Meddeb W., Nury T., Rezig L., Debbabi M., Bessam F.Z., Brahmi F., Vejux A., Mejri M., Martin C.R., Preedy V.R. (2020). Chapter 10—Antioxidant and neuroprotective properties of Mediterranean oils: Argan oil, olive oil, and milk thistle seed oil. Oxidative Stress and Dietary Antioxidants in Neurological Diseases.

[4] Tan W.Y.T., Young B.E., Lye D.C., Chew D.E.K., Dalan R. (2020). Statin use is associated with lower disease severity in COVID-19 infection. Sci. Rep..

[5] Dolatabadi J.E.N., Hamishehkar H., Eskandani M., Valizadeh H. (2014). Formulation, characterization and cytotoxicity studies of alendronate sodium-loaded solid lipid nanoparticles. Colloids Surf. B Biointerfaces.

[6] Nutan B., Chandel A.K.S., Biswas A., Kumar A., Yadav A., Maiti P., Jewrajka S.K. (2020). Gold Nanoparticle Promoted Formation and Biological Properties of Injectable Hydrogels. Biomacromolecules.

[7] Yang Z., Peng H., Wang W., Liu T. (2010). Crystallization behavior of poly(ε-caprolactone)/layered double hydroxide nanocomposites. J. Appl. Polym. Sci..

[8] Ahmed T.A., Badr-Eldin S.M., Ahmed O.A.A., Aldawsari H. (2018). Intranasal optimized solid lipid nanoparticles loaded in situ gel for enhancing trans-mucosal delivery of simvastatin. J. Drug Deliv. Sci. Technol..

[9] Thoniyot P., Tan M.J., Karim A.A., Young D.J., Loh X.J. (2015). Nanoparticle–Hydrogel Composites: Concept, Design, and Applications of These Promising, Multi-Functional Materials. Adv. Sci..

[10] Qindeel M., Ullah M.H., Fakhar-ud-Din, Ahmed N., Rehman A. (2020). Recent trends, challenges and future outlook of transdermal drug delivery systems for rheumatoid arthritis therapy. J. Control. Release.

[11] Xia M., Cheng Y., Meng Z., Jiang X., Chen Z., Theato P., Zhu M. (2015). A novel nanocomposite hydrogel with precisely tunable UCST and LCST. Macromol. Rapid Commun..

[12] Duan Y., Dhar A., Patel C., Khimani M., Neogi S., Sharma P., Siva Kumar N., Vekariya R.L. (2020). A brief review on solid lipid nanoparticles: Part and parcel of contemporary drug delivery systems. RSC Adv..

[13] Ma G., Lin W., Wang Z., Zhang J., Qian H., Xu L., Yuan Z., Chen S. (2016). Development of polypeptide-based zwitterionic amphiphilic micelles for nanodrug delivery. J. Mater. Chem. B.

[14] Narayanan S., Pavithran M., Viswanath A., Narayanan D., Mohan C.C., Manzoor K., Menon D. (2014). Sequentially releasing dual-drug-loaded PLGA-casein core/shell nanomedicine: Design, synthesis, biocompatibility and pharmacokinetics. Acta Biomater..

[15] Martínez Rivas C.J., Tarhini M., Badri W., Miladi K., Greige-Gerges H., Nazari Q.A., Galindo Rodríguez S.A., Román R.Á., Fessi H., Elaissari A. (2017). Nanoprecipitation process: From encapsulation to drug delivery. Int. J. Pharm..

[16] Tella A.C., Okoro H.K., Sokoya S.O., Adimula V.O., Olatunji S.O., Zvinowanda C., Ngila J.C., Shaibu R.O., Adeyemi O.G. (2020). Synthesis, Characterization and Antifungal Activity of Fe(III) Metal–Organic Framework and its Nano-Composite. Chem. Afr..

[17] Mohammadi-Samani S., Salehi H., Entezar-Almahdi E., Masjedi M. (2020). Preparation and characterization of sumatriptan loaded solid lipid nanoparticles for transdermal delivery. J. Drug Deliv. Sci. Technol..

[18] Alvarez-Figueroa M.J., Narváez-Araya D., Armijo-Escalona N., Carrasco-Flores E.A., González-Aramundiz J.V. (2020). Design of Chitosan Nanocapsules with Compritol 888 ATO^®^ for Imiquimod Transdermal Administration. Evaluation of Their Skin Absorption by Raman Microscopy. Pharm. Res..

[19] Eltellawy Y.A., El-Kayal M., Abdel-Rahman R.F., Salah S., Shaker D.S. (2021). Optimization of transdermal atorvastatin calcium–Loaded proniosomes: Restoring lipid profile and alleviating hepatotoxicity in poloxamer 407-induced hyperlipidemia. Int. J. Pharm..

[20] Luo Y., Teng Z., Li Y., Wang Q. (2015). Solid lipid nanoparticles for oral drug delivery: Chitosan coating improves stability, controlled delivery, mucoadhesion and cellular uptake. Carbohydr. Polym..

[21] Saqib M., Shabbir Ali Bhatti A., Ahmad N.M., Ahmed N., Shahnaz G., Lebaz N., Elaissari A. (2020). Amphotericin b loaded polymeric nanoparticles for treatment of leishmania infections. Nanomaterials.

[22] Rana I., Khan N., Ansari M.M., Shah F.A., Din F.U., Sarwar S., Imran M., Qureshi O.S., Choi H.I., Lee C.H. (2020). Solid lipid nanoparticles-mediated enhanced antidepressant activity of duloxetine in lipopolysaccharide-induced depressive model. Colloids Surf. B Biointerfaces.

[23] Harisa G.I., Alomrani A.H., Badran M.M. (2017). Simvastatin-loaded nanostructured lipid carriers attenuate the atherogenic risk of erythrocytes in hyperlipidemic rats. Eur. J. Pharm. Sci..

[24] Qindeel M., Ahmed N., Sabir F., Khan S., Ur-Rehman A. (2019). Development of novel pH-sensitive nanoparticles loaded hydrogel for transdermal drug delivery. Drug Dev. Ind. Pharm..

[25] Jana S., Manna S., Nayak A.K., Sen K.K., Basu S.K. (2014). Carbopol gel containing chitosan-egg albumin nanoparticles for transdermal aceclofenac delivery. Colloids Surf. B Biointerfaces.

[26] Zeb A., Qureshi O.S., Yu C.H., Akram M., Kim H.S., Kim M.S., Kang J.H., Majid A., Chang S.Y., Bae O.N. (2017). Enhanced anti-rheumatic activity of methotrexate-entrapped ultradeformable liposomal gel in adjuvant-induced arthritis rat model. Int. J. Pharm..

[27] Siddiqui B., ur.Rehman A., Haq I.-U., Ahmad N.M., Ahmed N. (2020). Development, optimisation, and evaluation of nanoencapsulated diacerein emulgel for potential use in osteoarthritis. J. Microencapsul..

[28] Seong J.S., Yun M.E., Park S.N. (2018). Surfactant-stable and pH-sensitive liposomes coated with *N*-succinyl-chitosan and chitooligosaccharide for delivery of quercetin. Carbohydr. Polym..

[29] Naz K., Shahnaz G., Ahmed N., Qureshi N.A., Sarwar H.S., Imran M., Khan G.M. (2017). Formulation and In Vitro Characterization of Thiolated Buccoadhesive Film of Fluconazole. AAPS PharmSciTech.

[30] Jamadar M.J., Shaikh R.H. (2017). Preparation and Evaluation of Herbal Gel Formulation. J. Pharaceutical Res. Educ..

[31] Qureshi O.S., Kim H.S., Zeb A., Choi J.S., Kim H.S., Kwon J.E., Kim M.S., Kang J.H., Ryou C., Park J.S. (2017). Sustained release docetaxel-incorporated lipid nanoparticles with improved pharmacokinetics for oral and parenteral administration. J. Microencapsul..

[32] Azeez L., Lateef A., Adejumo A.L., Adeleke J.T., Adetoro R.O., Mustapha Z. (2020). Adsorption Behaviour of Rhodamine B on Hen Feather and Corn Starch Functionalized with Green Synthesized Silver Nanoparticles (AgNPs) Mediated with Cocoa Pods Extracts. Chem. Afr..

[33] Dar M.J., Din F.U., Khan G.M. (2018). Sodium stibogluconate loaded nano-deformable liposomes for topical treatment of leishmaniasis: Macrophage as a target cell. Drug Deliv..

[34] Panchagnula R., Bokalial R., Sharma P., Khandavilli S. (2005). Transdermal delivery of naloxone: Skin permeation, pharmacokinetic, irritancy and stability studies. Int. J. Pharm..

[35] Giuliano E., Paolino D., Fresta M., Cosco D. (2018). Drug-Loaded Biocompatible Nanocarriers Embedded in Poloxamer 407 Hydrogels as Therapeutic Formulations. Medicines.

[36] Ahmed S., Gull A., Aqil M., Danish Ansari M., Sultana Y. (2019). Poloxamer-407 thickened lipid colloidal system of agomelatine for brain targeting: Characterization, brain pharmacokinetic study and behavioral study on Wistar rats. Colloids Surf. B Biointerfaces.

[37] Schröder A., Sprakel J., Schroën K., Spaen J.N., Berton-Carabin C.C. (2018). Coalescence stability of Pickering emulsions produced with lipid particles: A microfluidic study. J. Food Eng..

[38] Patil S.G., Jadhav V.M., Kadam V.J. (2012). Formulation and Evaluation of Herbal Gel. Indian J. Nat. Prod. Resour..

[39] De Mendoza A.E.H., Campanero M.A., Mollinedo F., Blanco-Prieto M.J. (2009). Lipid nanomedicines for anticancer drug therapy. J. Biomed. Nanotechnol..

[40] Qureshi O.S., Zeb A., Akram M., Kim M.S., Kang J.H., Kim H.S., Majid A., Han I., Chang S.Y., Bae O.N. (2016). Enhanced acute anti-inflammatory effects of CORM-2-loaded nanoparticles via sustained carbon monoxide delivery. Eur. J. Pharm. Biopharm..

[41] Dudhipala N., Veerabrahma K. (2017). Improved anti-hyperlipidemic activity of Rosuvastatin Calcium via lipid nanoparticles: Pharmacokinetic and pharmacodynamic evaluation. Eur. J. Pharm. Biopharm..

[42] Jaafar-Maalej C., Diab R., Andrieu V., Elaissari A., Fessi H. (2010). Ethanol injection method for hydrophilic and lipophilic drug-loaded liposome preparation. J. Liposome Res..

[43] Tarhini M., Benlyamani I., Hamdani S., Agusti G., Fessi H., Greige-Gerges H., Bentaher A., Elaissari A. (2018). Protein-based nanoparticle preparation via nanoprecipitation method. Materials.

[44] Md S., Kuldeep Singh J.K.A., Waqas M., Pandey M., Choudhury H., Habib H., Hussain F., Hussain Z. (2019). Nanoencapsulation of betamethasone valerate using high pressure homogenization–solvent evaporation technique: Optimization of formulation and process parameters for efficient dermal targeting. Drug Dev. Ind. Pharm..

[45] Jiang T., Han N., Zhao B., Xie Y., Wang S. (2012). Enhanced dissolution rate and oral bioavailability of simvastatin nanocrystal prepared by sonoprecipitation. Drug Dev. Ind. Pharm..

[46] Rauf A., Razzaq S., Tabish T.A., Tahseen S., Sandhu M.A., Shahnaz G. (2021). Investigating the intracellular bactericidal effects of rifampicin loaded S-protected thiomeric chitosan nanocargoes against *Mycobacterium tuberculosis*. J. Drug Deliv. Sci. Technol..

[47] Hasan A.S., Socha M., Lamprecht A., Ghazouani F., El Sapin A., Hoffman M., Maincent P., Ubrich N. (2007). Effect of the microencapsulation of nanoparticles on the reduction of burst release. Int. J. Pharm..

[48] Chen H., Chang X., Du D., Li J., Xu H., Yang X. (2006). Microemulsion-based hydrogel formulation of ibuprofen for topical delivery. Int. J. Pharm..

[49] Gangurde A.B., Amin P.D. (2017). Microencapsulation by Spray Drying of Vitamin A Palmitate from Oil to Powder and Its Application in Topical Delivery System. J. Encapsul. Adsorpt. Sci..

[50] Li X., Yang Q., Zhao Y., Long S., Zheng J. (2017). Dual physically crosslinked double network hydrogels with high toughness and self-healing properties. Soft Matter.

[51] Mirzaei B.E., Ramazani A., Shafiee M., Danaei M. (2013). Studies on glutaraldehyde crosslinked chitosan hydrogel properties for drug delivery systems. Int. J. Polym. Mater. Polym. Biomater..

[52] Puri A., Frempong D., Mishra D., Dogra P. (2021). Microneedle-mediated transdermal delivery of naloxone hydrochloride for treatment of opioid overdose. Int. J. Pharm..

[53] Din F.U., Saleem S., Aleem F., Ahmed R., Huda N.U., Ahmed S., Khaleeq N., Shah K.U., Ullah I., Zeb A. (2018). Advanced colloidal technologies for the enhanced bioavailability of drugs. Cogent Med..

[54] Kawar D., Abdelkader H. (2019). Hyaluronic acid gel-core liposomes (hyaluosomes) enhance skin permeation of ketoprofen. Pharm. Dev. Technol..

[55] Khalid A., Ahmed N., Qindeel M., Asad M.I., Khan G.M., Rehman A.U. (2021). Development of novel biopolymer-based nanoparticles loaded cream for potential treatment of topical fungal infections. Drug Dev. Ind. Pharm..

